# Image quality and pathology assessment in CT Urography: when is the low-dose series sufficient?

**DOI:** 10.1186/s12880-019-0363-z

**Published:** 2019-08-09

**Authors:** Bharti Kataria, Jonas Nilsson Althén, Örjan Smedby, Anders Persson, Hannibal Sökjer, Michael Sandborg

**Affiliations:** 1 Department of Radiology, Linköping University, Linköping, Sweden; 2 Department of Medical & Health Sciences, Linköping University, Linköping, Sweden; 3 Center for Medical Image Science & Visualization (CMIV), Linköping University, Linköping, Sweden; 4 Department of Medical Physics, Linköping University, Linköping, Sweden; 5 Department of Biomedical Engineering and Health Systems (MTH), KTH Royal Institute of Technology, Stockholm, Sweden

**Keywords:** Computed tomography, Urography, Low-dose, Optimization, Image quality, Dose

## Abstract

**Background:**

Our aim was to compare CT images from native, nephrographic and excretory phases using image quality criteria as well as the detection of positive pathological findings in CT Urography, to explore if the radiation burden to the younger group of patients or patients with negative outcomes can be reduced.

**Methods:**

This is a retrospective study of 40 patients who underwent a CT Urography examination on a 192-slice dual source scanner. Image quality was assessed for four specific renal image criteria from the European guidelines, together with pathological assessment in three categories: renal, other abdominal, and incidental findings without clinical significance. Each phase was assessed individually by three radiologists with varying experience using a graded scale. Certainty scores were derived based on the graded assessments. Statistical analysis was performed using visual grading regression (VGR). The limit for significance was set at *p* = 0.05.

**Results:**

For visual reproduction of the renal parenchyma and renal arteries, the image quality was judged better for the nephrogram phase (*p* < 0.001), whereas renal pelvis/calyces and proximal ureters were better reproduced in the excretory phase compared to the native phase (*p* < 0.001). Similarly, significantly higher certainty scores were obtained in the nephrogram phase for renal parenchyma and renal arteries, but in the excretory phase for renal pelvis/calyxes and proximal ureters. Assessment of pathology in the three categories showed no statistically significant differences between the three phases. Certainty scores for assessment of pathology, however, showed a significantly higher certainty for renal pathology when comparing the native phase to nephrogram and excretory phase and a significantly higher score for nephrographic phase but only for incidental findings.

**Conclusion:**

Visualisation of renal anatomy was as expected with each post-contrast phase showing favourable scores compared to the native phase. No statistically significant differences in the assessment of pathology were found between the three phases. The low-dose CT (LDCT) seems to be sufficient in differentiating between normal and pathological examinations. To reduce the radiation burden in certain patient groups, the LDCT could be considered a suitable alternative as a first line imaging method. However, radiologists should be aware of its limitations.

**Electronic supplementary material:**

The online version of this article (10.1186/s12880-019-0363-z) contains supplementary material, which is available to authorized users.

## Background

CT Urography (CTU) has emerged as the modality of choice in imaging of the abdomen in patients with urinary tract diseases due to its high sensitivity and specificity [1]. Although it comes with a high radiation dose penalty, the benefits of CT imaging outweigh the risk for many of these patients. Optimisation is not only about patient dose and image quality but also about the diagnostic task at hand, i.e. the correct examination technique for a specific diagnostic enquiry in accordance to the ALARA (radiation dose as low as reasonably achievable) and AHARA (image quality as high as reasonably achievable) principles [2, 3]. Patients presented with haematuria or acute flank pain usually undergo diagnostic imaging to rule out any serious conditions underlining upper urinary tract disease such as urolithiasis, renal cell cancer or upper urinary tract urothelial cell carcinoma (UUT-UCC) [4]. Urolithiasis is a common health problem with a high recurrent rate requiring considerable radiological imaging resources for this population, many of which are younger than 50 years of age [5].

The standardized care pathway (SCP) led to general recommendations of the use of medical imaging in diagnostics of urinary tract disease for patients with macroscopic hematuria who are ≥40 years (revised to ≥50 years in 2018), but even younger patients with risk factors are investigated [6]. The majority of the SCP population consist of malignant cancer diagnoses of the urinary tract and a third of the patients present with benign causes of hematuria [6]. Approximately 20–30% of these patients with symptoms of visible blood have negative outcomes and are being subjected to diagnostic imaging tests and radiation related risks based on the presence of macro-hematuria [6].

Macroscopic hematuria is a common symptom in other treatable benign diseases such as urinary tract infection and targets younger women of child bearing age, among others [7].

CTU is a multiphase examination associated with a relatively high radiation dose and can be justified as a first line investigation in hematuria patients if important risk factors are present and clinical tests indicate high risk probability of cancer [8]. In the emergency department, where the short examination times as well as the detection of alternative diagnoses is paramount, CT is the preferred modality with a selection of scan protocols depending on the indication. The detrimental effects of ionizing radiation and the expanding use of CT technique have been the driving factors in optimization of clinical practices regarding appropriateness criteria and dose reduction [9]. These can be achieved foremost by selective use of nonradiative modalities such as ultrasound and magnetic resonance imaging (MRI) and referrals based on proper clinical indications, preliminary tests and patient groups. Dose reduction is achieved in a number of ways, such as, limiting scan lengths and number of phases, and the use of lower exposure settings. Dose reduction is additionally seen in the use of features such as automatic dose modulation, iterative reconstruction [10–12] and split-bolus techniques especially in diagnostic imaging of younger, high risk patients [13, 14]. Recent advancements show a trend towards use of low-dose CT (LDCT) in several diagnostic indications such as the investigation of acute flank pain and acute abdomen for diverticulitis, appendicitis and renal stone disease [15, 16].

Both the Bonn call for action and the triple AAA campaign were introduced to strengthen the need for stringent measures in radiation protection for safe and appropriate use of ionizing radiation in medical imaging [17–19]. Published literature on renal stone evaluation have validated the trend towards use of low-dose CT (LDCT) due to the high contrast between urinary stones and the surrounding soft tissue [15, 20] as well as in investigation of acute abdomen [16]. However, implementation of the LDCT protocol in the clinical setting has been very slow partly due to the low quality of the images and lack of confidence in interpreting reduced-dose images [21]. But with practice and growing experience it is possible to increase diagnostic confidence and acceptance of lower quality images [22].

The diagnostic performance of non-enhanced CT compared to intravenous urography (IVU) [23] and plain abdominal radiography [24] has been evaluated but there are, to our knowledge, no studies that have compared the image quality and pathology assessment between phases as the present study.

The aim was to compare CT images from native, nephrographic and excretory phases using image quality criteria as well as the detection of positive pathological findings in CT Urography to explore if the radiation burden to the younger group of patients, patients undergoing repetitive imaging or patients with negative outcomes can be reduced.

## Materials & methods

This is a retrospective study approved by the regional ethical board. Of the 50 patients referred for a clinical CTU between 2016-03-14 and 2016-11-22 and examined on a 192-slice dual source scanner in single source mode (Siemens Healthineers, Erlangen, Germany), forty patients were included in the study. The acquisition data are presented in Table 1.

**Table 1:** Acquisition parameters for a clinical CT Urography on a 192-slice dual source scanner in a single source scan mode

Phase	Acquisition data	kV	Qref mAs	Gantry Rotation (s)	Pitch	Slice thickness/increment mm	Scan Delay	Care kV
Native	192 × 0.6	120	45	0.5	0.6	3/2		On
Nephrogram	192 × 0.6	120	140	0.5	0.6	3/2	100 s	Semi
Excretory	192 × 0.6	120	45	0.5	0.6	3/2	10 min	On

The scan length ranged from diaphragm to symphysis pubis. The images were reconstructed with kernel Bf36 and iterative reconstruction ADMIRE strength 3. In the nephrographic phase semi Care kV setting was used resulting in 100 kV and effective tube load of 181mAs

Ten patients were excluded due to motion artefacts, difference in scan protocol and scan range. The standard CTU protocol was used with intravenous administration of contrast medium, Iopromide (Ultravist 370mgI/ml, Bayer, Dublin, Ireland), the rate and dose tailored to patient body weight using OMNIVIS calculator (GE healthcare). Computed Tomography Dose Index (CTDI_vol_) and, Dose Length Product (DLP) were recorded and Size Specific Dose Estimate (SSDE) was calculated based on the antero-posterior (AP) and lateral (LAT) dimensions of each patient at the level of the kidneys using the center slice approach as described in Boos et al. [25, 26].

**Fig. 1:**
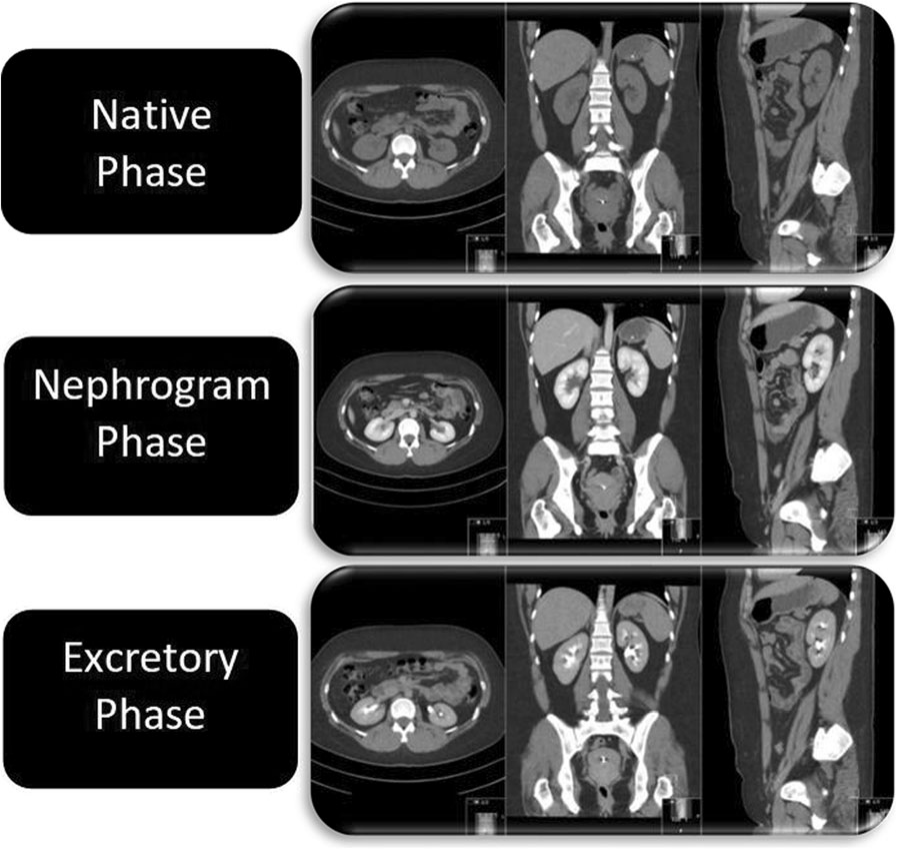
Illustration of compared phases reconstructed with multi-planar reconstruction (MPR) in three planes. The images are from a CT Urography examination of a study patient

The standard protocol for CTU consists of three phases: the native or unenhanced phase (low-dose), a nephrographic phase (standard dose) and an excretory phase (low-dose), all of which are reconstructed with multi-planar reconstruction (MPR) in three planes: axial, coronal and sagittal (Fig. 1). In patients < 50 years of age, the nephrographic phase is limited to the upper abdomen. Patients > 50 years of age are examined with all three phases from dome of diaphragm to symphysis pubis arch.

During the reading sessions the readers were asked to grade four anatomical structures (renal parenchyma, renal pelvis and calyxes, proximal ureters and renal arteries) obtained from European guidelines for quality criteria on a five-point Likert scale with numerical scores from one to five allocated to response alternatives; criterion was fulfilled, criterion was probably fulfilled, indecisive, criterion was probably not fulfilled and criterion was not fulfilled (Table 2).

**Table 2:** Anatomical image criteria assessed for each phase including axial, coronal and sagittal multiplanar reconstruction (MPR) planes and graded on a 5-point Likert type scale by allocating a score of 1 to 5

Image Criteria	
C1: Visually sharp reproduction of the renal parenchyma	
C2: Visually sharp reproduction of the renal pelvis and calyxes	
C3: Visually sharp reproduction of the proximal part of the ureters	
C4: Visually sharp reproduction of the renal arteries	
Grading Scale scores	
1: Criterion was fulfilled	
2: Criterion was probably fulfilled	
3: Indecisive	
4: Criterion was probably not fulfilled	
5: Criterion was not fulfilled	

The readers were also asked to assess the presence of pathology in three categories; renal, abdominal and incidental findings. These were also graded on a five-point Likert-type scale based on numerical scores from one to five with response alternatives; normal examination, probably normal examination, indecisive, probably pathological examination and pathological examination (Table 3). To determine how certain the readers were in their scoring of the image criteria and pathological findings, variation in certainty scores between the three phases were also calculated by grouping the score options as shown in Table 4.

**Table 3:** Pathology categories were assessed for each phase including axial, coronal and sagittal multiplanar reconstruction (MPR) planes and graded on a 5-point Likert type scale by allocating a score of 1 to 5

Pathology categories	
C5: Pathology in the kidneys and urinary tract related to abdominal symptoms	
C6: Other pathology related to abdominal symptoms	
C7: Incidental findings without clinical significance	
Grading Scale scores	
1: Normal examination	
2: Probably normal examination	
3: Inconclusive examination	
4: Probably pathological examination	
5: Pathological examination

**Table 4:** Certainty scores obtained by grouping the assessment scores for anatomical criteria and pathology

	Grouping of scores
Certainty score: Image criteria	
High	1 and 5 (was fulfilled/not fulfilled)
Medium	2 and 4 (probably fulfilled/probably not fulfilled)
Low	3 (indecisive)
Certainty score: Pathology	
High	1 and 5 (normal examination/pathological examination)
Medium	2 and 4 (probably normal examination/probably pathological examination)
Low	3 (indecisive)

Statistical analysis within the Visual Grading Regression (VGR) framework [27] was performed with the software Stata 13.1 (Stata Corporation LP, College Station, TX, USA) using the multi-level mixed-effects ordered logistic regression (*meologit)* command for image quality scores [28]. Thus, ordinal logistic regression was applied to scores from observer ratings whilst controlling for dependencies between observers and patients, where the regression coefficients describe the combined effect of dose and intravenous contrast on image quality, related to each of the phases of the CTU. The certainty scores were analyzed using the same statistic model as the original scores.

## Results

Of the 40 patients included in the study, 12 were women, age range 27–74 years (mean 58.9 ± 14 (SD)) with a body mass index (BMI) of 20.7–35.7 kg/m^2^ (mean 27.7 ± 5.0) and 28 men, age range 29–85 years (mean 63.8 ± 15.0) with a BMI of 19.3–38.9 kg/m^2^ (mean 27.0 ± 3.7). The mean values, SD and ranges of DLP, CTDI_vol,_ SSDE for the native, nephrogram and excretory phases are displayed in Table 5.

**Table 5:** Distribution of ranges, standard deviation (SD) and mean values of dose length product (DLP), Computed Tomography Dose Index (CTDI_vol_) and size specific dose estimate (SSDE)

Phase	DLP mGy ^.^cm	CTDI_vol_ mGy	SSDE mGy
Native			
Mean	135.7	2.84	3.51
SD	38.3	0.73	0.79
Range	63.8–257.6	1.45–5.39	2.28–6.93
Nephrogram			
Mean	336.2	6.9	8.52
SD	100.6	1.9	2.55
Range	151.5–696.8	3.46–14.58	5.45–18.75
Excretory			
Mean	135.4	2.83	3.49
SD	38.7	0.73	0.79
Range	65.4–254.0	1.49–5.32	2.29–6.84

### Assessment of anatomical image criteria

**Fig. 2:**
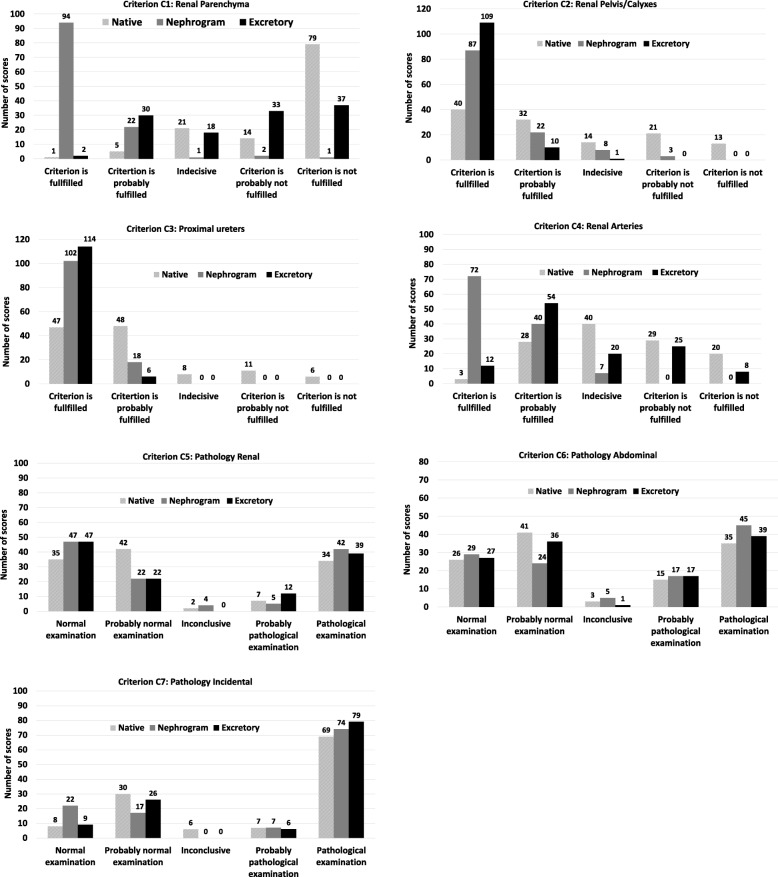
Distribution of number of scores (annotation above each bar) allocated to each criterium assessed by readers in a Computed Tomography Urography (CTU). All differences between phases are significant for Criterion 1 to 4. All differences between phases were non-significant for Criterion C5 to C7 (Additional file 1: Table S1)

When comparing the native phase with nephrogram and excretory phase respectively, criteria C1 and C4 (renal parenchyma and renal arteries) were better reproduced in the nephrographic phase, whereas criteria C2 and C3 (renal pelvis/calyxes, proximal ureters), were visually better reproduced in the excretory phase, (*p* < 0.001). Similar results were obtained when comparing the nephrogram to excretory phase, where the nephrographic phase was favorable for criteria C1 (renal parenchyma) and C4 (renal arteries) (*p* < 0.001). For the delineation of criteria C2 (renal pelvis/calyxes) and C3 (proximal ureters), the excretory phase was preferred (*p* < 0.001 and *p* = 0.034, respectively) (Fig. 2).

### Assessment of pathology

For the detection of renal and other abdominal pathologies, only marginal and not statistically significant differences in scores were found when comparing the native phase with nephrographic and excretory phases. Similarly, the scores for incidental findings in all three phases were not significantly different. For all three pathology categories, the number of inconclusive scores were very low suggesting that normality and pathology could be assessed in all three phases with marginal differences in scores. The native phase had a higher number of probably normal examination scores compared to the other two phases. None of these differences were statistically significant.

### Certainty scores

#### Image criteria

High certainty scores are seen for criteria C1 (renal parenchyma) and C4 (renal arteries) in favor of the nephrographic phase, when comparing native to nephrographic phase (*p* < 0.001). Similar results are seen for comparison between nephrographic and excretory phase with significant differences in favor of the nephrographic phase (*p* < 0.001). However, when comparing native and excretory phases, certainty for criterion C1 (renal parenchyma) was statistically significantly higher for the native phase whereas no significant difference was found for criterion C4 (renal arteries). Criteria C2 (renal pelvis/calyxes) and C3 (proximal ureters) showed significantly higher certainty scores in the contrast-enhanced phases when comparing native phase to nephrographic and excretory phases (*p* < 0.001). Comparisons between nephrographic and excretory phases show a highly significant difference in scores in favor of excretory phase for criterion C2 (renal pelvis/calyxes) (*p* < 0.001) and a significant difference in scores for criterion C3 (proximal ureters) (*p* = 0.021).

#### Pathology

Significantly high certainty scores in favor of the contrast enhanced phases are obtained for C5 (renal pathology) (*p* < 0.05), when comparing native to nephrographic and excretory phases, however, there was no significant difference between the two contrast-enhanced phases. Marginal not significant differences in medium and high certainty scores are observed for Criteria C6 (abdominal pathology) and C7 (incidental findings) when comparing all three phases with each other. Except for determination of C7 (incidental findings), which was statistically significant in favor of the nephrographic phase (*p* < 0.01), when native and nephrographic phases were compared, all other differences in scores between phases were not significant.

## Discussion

**Fig. 3:**
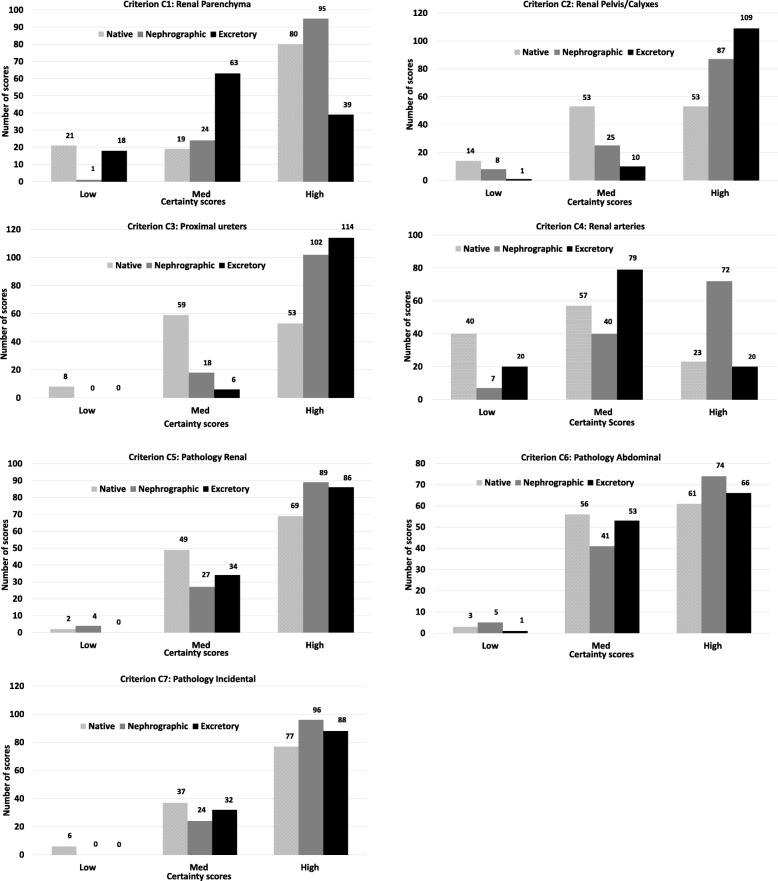
Distribution of number of certainty scores (annotation above each bar) allocated to each criterion assessed by readers in a Computed Tomography Urography (CTU) protocol. The scores 1 and 5 were grouped for high certainty, scores 2 and 4 for medium certainty and a score of 3 for low certainty. Significance values are presented in supplementary data (Additional file 2: Table S2)

The present study showed that the contrast-enhanced phases were considered significantly better for determination of renal pathology. This is not an unusual finding as the acquisition delays after contrast injection are designed to render anatomical features in the best possible way. However, the marginal differences in scores when assessing pathology in the three categories were not significant, with very few inconclusive scores suggesting that it was possible to determine whether the examination was normal or pathological in all three phases. This is also demonstrated by the larger number of high and medium certainty scores for all three categories (Fig. 3). The LDCT is one of many alternatives, that could possibly be used to differentiate between normal and pathological in order to reduce the radiation burden in patients with negative outcomes and those who are more sensitive to ionising radiation.

There are studies that have evaluated the effect of iterative reconstruction on dose reduction in patients presented with acute abdominal pain [11, 12]. Lee et al. [12] concluded that despite subjective differences in image quality between full-dose and half-dose images, the diagnostic performance is maintained for the low-dose images with the exception of lesion detection at sub-centimeter size. Poletti et al. [11] concluded that low-dose imaging can be achieved in non-obese patients. However, these studies were performed with contrast enhancement which, combined with dose reduction properties of IR, maintains the diagnostic performance of the low-dose protocol when compared to contrast-enhanced standard-dose protocol.

In order to optimize the use of CT in urinary tract investigations, there are several methods that can be used to reduce dose in abdominal CT such as modifying scanning parameters [29], using a combination of standard-dose and low-dose phases in a CTU protocol [30] and reducing the number of acquisitions and scan lengths [14]. As the implementation of the LDCT has been very slow, this study provides an insight into the possible applicability of this protocol in clinical practice to reduce radiation dose for adolescents and children, as well as patients that require repetitive imaging.

Our CTU protocol is optimized using automatic dose modulation as well as iterative reconstruction and combination of low-dose and standard-dose series in concurrence with Dahlman et al. [30] who obtained significant dose reductions, in the unenhanced and excretory phases, achieved when combined with one normal-dose phase. However, the dilemma of reducing the radiation burden in certain patient groups still remains. Some institutions have adopted a work-flow routine in order to minimize the number of phases required by viewing the low-dose series to determine further need for imaging (Magnusson A, CT lecture, Larvik, 2018, personal communications). However due to logistics this is not always possible especially for referrals from the emergency department where time is of essence.

One of the sites at our institution extensively adopted the LDCT in 2012, to meet the urologists needs for imaging after Extracorporal Shockwave Lithotripsy (ESWL) as well as follow-up imaging of patients with nephro- and uro-lithiasis. With growing expertise and increasing diagnostic confidence, the comfort zone boundaries were broadened and the LDCT use was extended to diagnostics of acute flank pain and acute abdomen for several indications such as diverticulitis, appendicitis and renal calculi [22] in concurrence with Hamimi et al. [31] who studied the efficiency of low-dose technique using an effective mAs of 50 (very similar to our LDCT protocol), in diagnosis of renal calculi and concluded that it was crucial in the management of renal stone disease in the acute setting. Both Lee et al. [12] and Poletti et al. [11] demonstrated the use of LDCT in acute abdominal pain diagnoses. An LDCT protocol can be considered as a valuable tool in acute abdominal pain evaluation as it allows for many possible differential diagnoses, but radiologists should also be aware of its limitations [22].

The statistical method (VGR) that we used, allowed us to analyze the three phases in the same analysis, with subsequent pair-wise comparisons. VGR also has the ability to let the researcher estimate the potential dose reduction resulting from changes in the imaging protocol. However, the design of this retrospective study does not permit such an analysis.

There is one major study limitation: due to the retrospective nature of our study we failed to separate the individual effects of dose and contrast enhancement on image quality. Ideally to determine these effects individually, all three phases should have been compared at different dose levels which would also allow for estimation of the potential dose reduction for each individual phase [32]. However, this would have increased the radiation burden for these patients.

## Conclusion

Visualisation of renal anatomy in the three phases were as expected with each post-contrast phase showing favourable scores compared to the native phase. No statistically significant differences in the assessment of pathology were found between the three phases.

Since many certainty scores were in the high and medium categories, the LDCT seems to be sufficient to differentiate between normal and pathological examinations. In order to reduce the radiation burden in adolescents and children, as well as patients with negative outcomes and those that require repetitive imaging, the LDCT could be considered a suitable alternative as a first line imaging method. However, radiologists should be aware of its limitations.

## Additional files

Additional file 1: **Table S1.** Scores for each of the criteria. Significance of differences between phases tested with mixed-effects ordinal logistic regression, with pairwise comparisons using Bonferroni correction. (DOCX 63 kb)

Additional file 2: **Table S2.** Certainty scores for each of the criteria. Significance of differences between phases tested with mixed-effects ordinal logistic regression in phase comparisons using Bonferroni correction. (DOCX 55 kb)

## Data Availability

The datasets used and/ or analyzed during the current study are available from the corresponding author on reasonable request.

## Abbreviations

AHARA: As high as reasonably achievable
ALARA: As low as reasonably achievable
CTDI_vol_: Computed Tomography Dose Index
CTU: Computed Tomography Urography
DLP: Dose Length Product
ESWL: Extracorporal Shock Wave Lithotripsy
kV: kilovolt
LDCT: Low-dose Computed Tomography
mAs: milliampere seconds
mGy: milligray
MRI: Magnetic Resonance Imaging
mSv: millisievert
SCP: Standardized Care Pathway
SD: Standard deviation
SSDE: Size Specific Dose Estimate
