# Supplementary material for: Image quality and pathology assessment in CT Urography: when is the low-dose series sufficient?
Source: BMC Med Imaging. 2019 Aug 9;19:64. doi: 10.1186/s12880-019-0363-z (PMC6688276; doi:10.1186/s12880-019-0363-z)
Supplement: Supplementary file 1 — Table S1. Scores for each of the criteria. Significance of differences between phases tested with mixed-effects ordinal logistic regression, with pairwise comparisons using Bonferroni correction. (DOCX 63 kb) [file 12880_2019_363_MOESM1_ESM.docx]

Additional file 1: **Table S1**. Scores for each of the criteria. Significance of differences between phases tested with mixed-effects ordinal logistic regression, with pairwise comparisons using Bonferroni correction.

|  |  | **Scores** | | | | | | | | |  | **Significance tests (p value) phase comparisons** | | | |  |
| --- | --- | --- | --- | --- | --- | --- | --- | --- | --- | --- | --- | --- | --- | --- | --- | --- |
| **Criterion** | **Phase** | **1** | **2** | | **3** | | | **4** | | **5** | **Total** | **overall** | **1 vs. 2** | **1 vs. 3** | **2 vs. 3** |  |
| C1 Renal parenchyma | 1. Native | 1 | 5 | | 21 | | | 14 | | 79 | 120 | <0.001 | <0.001 | <0.001 | <0.001 |  |
|  | 2. Nephrographic | 94 | 22 | | 1 | | | 2 | | 1 | 120 |  |  |  |  |  |
|  | 3. Excretory | 2 | 30 | | 18 | | | 33 | | 37 | 120 |  |  |  |  |  |
|  | | | | | | | | | | | | | | | | |
| C2 Renal pelvis/calyxes | 1. Native | 40 | 32 | | 14 | | | 21 | | 13 | 120 | <0.001 | <0.001 | <0.001 | <0.001 |  |
|  | 2. Nephrographic | 87 | 22 | | 8 | | | 3 | | 0 | 120 |  |  |  |  |  |
|  | 3. Excretory | 109 | 10 | | 1 | | | 0 | | 0 | 120 |  |  |  |  |  |
|  | | | | | | | | | | | | | | | | |
| C3 Proximal ureters | 1. Native | 47 | 48 | | 8 | | | 11 | | 6 | 120 | <0.001 | <0.001 | <0.001 | <0.05  (0.034) |  |
|  | 2. Nephrographic | 102 | 18 | | 0 | | | 0 | | 0 | 120 |  |  |  |  |  |
|  | 3. Excretory | 114 | 6 | | 0 | | | 0 | | 0 | 120 |  |  |  |  |  |
|  | | | | | | | | | | | | | | | | |
| C4 Renal arteries | 1. Native | 3 | 28 | | 40 | | | 29 | | 20 | 120 | <0.001 | <0.001 | <0.001 | <0.001 |  |
|  | 2. Nephrographic | 72 | 40 | | 7 | | | 0 | | 0 | 120 |  |  |  |  |  |
|  | 3. Excretory | 12 | 54 | | 20 | | | 25 | | 8 | 120 |  |  |  |  |  |
|  | | | | | | | | | | | | | | | | |
| C5 Renal pathology | 1. Native | 35 | 42 | | 2 | | | 7 | | 34 | 120 | n.s. | n.s  (1.000) | n.s (1.000) | n.s.  (1.000) |  |
|  | 2. Nephrographic | 47 | 22 | | 4 | | | 5 | | 42 | 120 |  |  |  |  |  |
|  | 3. Excretory | 47 | 22 | | 0 | | | 12 | | 39 | 120 |  |  |  |  |  |
|  | | | | | | | | | | | | | | | | |
| C6 Other abdominal pathology | 1. Native | 26 | 41 | | 3 | | | 15 | | 35 | 120 | n.s. | n.s.  (0.512) | n.s (1.000) | n.s.  (0.974) |  |
|  | 2. Nephrographic | 29 | 24 | | 5 | | | 17 | | 45 | 120 |  |  |  |  |  |
|  | 3. Excretory | 27 | 36 | | 1 | | | 17 | | 39 | 120 |  |  |  |  |  |
|  | | | | | | | | | | | | | | | | |
| C7 Incidental pathology | 1. Native | 8 | 30 | | 6 | | | 7 | | 69 | 120 | n.s. | n.s.  (0.892) | n.s (0.802) | n.s.  (0.111) |  |
|  | 2. Nephrographic | 22 | 17 | | 0 | | | 7 | | 74 | 120 |  |  |  |  |  |
|  | 3. Excretory | 9 | 26 | | 0 | | | 6 | | 79 | 120 |  |  |  |  |  |
|  |  |  | |  | |  |  | |  | |  |  |  |  |  |  |
