# Supplementary material for: Image quality and pathology assessment in CT Urography: when is the low-dose series sufficient?
Source: BMC Med Imaging. 2019 Aug 9;19:64. doi: 10.1186/s12880-019-0363-z (PMC6688276; doi:10.1186/s12880-019-0363-z)
Supplement: Supplementary file 2 — Table S2. Certainty scores for each of the criteria. Significance of differences between phases tested with mixed-effects ordinal logistic regression in phase comparisons using Bonferroni correction. (DOCX 55 kb) [file 12880_2019_363_MOESM2_ESM.docx]

**Additional file 2: Table S2.** Certainty scores for each of the criteria. Significance of differences between phases tested with mixed-effects ordinal logistic regression in phase comparisons using Bonferroni correction.

| **Criterion** | **Phase** | **Low (3)** | **Med**  **(2 or 4)** | **High**  **(1 or 5)** | **Total** | **Significance tests (p value) phase comparisons** | | | | |
| --- | --- | --- | --- | --- | --- | --- | --- | --- | --- | --- |
|  |  |  |  |  |  | **overall** | **1 vs. 2** | **1 vs. 3** | **2 vs. 3** |  |
| C1 Renal parenchyma | 1. Native | 21 | 19 | 80 | 120 | <0.001 | <0.001 | <0.001 | <0.001 |  |
|  | 2. Nephrographic | 1 | 24 | 95 | 120 |  |  |  |  |  |
|  | 3. Excretory | 18 | 63 | 39 | 120 |  |  |  |  |  |
|  | | | | | | | | | |  |
| C2 Renal pelvis/calyxes | 1. Native | 14 | 53 | 53 | 120 | <0.001 | <0.001 | <0.001 | <0.001 |  |
|  | 2. Nephrographic | 8 | 25 | 87 | 120 |  |  |  |  |  |
|  | 3. Excretory | 1 | 10 | 109 | 120 |  |  |  |  |  |
|  | | | | | | | | | |  |
| C3 Proximal ureters | 1. Native | 8 | 59 | 53 | 120 | <0.001 | <0.001 | <0.001 | 0.021 |  |
|  | 2. Nephrographic | 0 | 18 | 102 | 120 |  |  |  |  |  |
|  | 3. Excretory | 0 | 6 | 114 | 120 |  |  |  |  |  |
|  | | | | | | | | | |  |
| C4 Renal arteries | 1. Native | 40 | 57 | 23 | 120 | <0.001 | <0.001 | n.s.  (0.178) | <0.001 |  |
|  | 2. Nephrographic | 7 | 40 | 72 | 120 |  |  |  |  |  |
|  | 3. Excretory | 20 | 79 | 20 | 120 |  |  |  |  |  |
|  | | | | | | | | | |  |
| C5 Renal pathology | 1. Native | 2 | 49 | 69 | 120 | <0.05 | <0.05 (0.028) | <0.05  (0.046) | n.s |  |
|  | 2. Nephrographic | 4 | 27 | 89 | 120 |  |  |  |  |  |
|  | 3. Excretory | 0 | 34 | 86 | 120 |  |  |  |  |  |
|  | | | | | | | | | |  |
| C6 Other abdominal pathology | 1. Native | 3 | 56 | 61 | 120 | n.s. | n.s.  (0.369) | n.s.  (1.000) | n.s.  (1.000) |  |
|  | 2. Nephrographic | 5 | 41 | 74 | 120 |  |  |  |  |  |
|  | 3. Excretory | 1 | 53 | 66 | 120 |  |  |  |  |  |
|  | | | | | | | | | |  |
| C7 Incidental pathology | 1. Native | 6 | 37 | 77 | 120 | <0.01 | <0.01 (0.002) | n.s.  (0.071) | n.s.  (0.581) |  |
|  | 2. Nephrographic | 0 | 24 | 96 | 120 |  |  |  |  |  |
|  | 3. Excretory | 0 | 32 | 88 | 120 |  |  |  |  |  |
